# Phosphatidylserine Blockade Limits Systemic Dissemination of *Leishmania Amazonensis* Amastigotes

**DOI:** 10.1155/jimr/4561938

**Published:** 2026-10-19

**Authors:** Arieli Bernardo Portugal, Suzana Passos Chaves, Renato Augusto DaMatta, João Luiz Mendes Wanderley

**Affiliations:** ^1^ Immunoparasitology Laboratory Medical Sciences Institute Federal University of Rio de Janeiro Macaé RJ Brazil ufrj.br; ^2^ Cell and Tissue Biology Laboratory North Fluminense State University Campos Dos Goytacazes RJ Brazil

**Keywords:** dissemination, *L. amazonensis*, leishmaniasis, lesions, phosphatidylserine, visceralization

## Abstract

*Leishmania amazonensis* is associated with cases of cutaneous, disseminated, diffuse, mucosal, and visceral leishmaniasis (VL). The mechanisms underlying its ability to persist and spread after the initial clinical cure are unclear, although intrinsic characteristics of the parasite and the host may be involved. Phosphatidylserine (PS) is a pathogenic factor in both experimental models and in patients. It is related to macrophage infection, regulation of inflammatory and immune responses, and the extent and severity of the clinical outcomes of the disease. In a murine model of systemic infection, treatment with PS‐targeting antagonist antibodies significantly reduced parasite burden in the spleen. In parallel, the number and size of disseminated skin lesions were consistently reduced. These effects correlated with a substantial decrease in interleukin‐10 (IL‐10) and transforming growth factor‐β (TGF‐β) production by splenocytes stimulated with leishmanial antigens, indicating a reversal of the regulatory immune profile commonly associated with chronic infection. These findings support a critical role for PS in the dissemination and visceralization of *Leishmania amazonensis*, highlighting its contribution to immune evasion and the pathogenesis of severe disease forms.

## 1. Introduction

Leishmaniasis is a neglected tropical disease endemic to most tropical and subtropical regions. It is estimated that ~1 billion people live in areas at risk, with around 30,000 new cases of visceral leishmaniasis (VL) and over 1 million new cases of cutaneous leishmaniasis (CL) reported annually. The disease encompasses a broad spectrum of clinical manifestations, including VL, CL, mucosal leishmaniasis (MCL), diffuse CL (DCL), disseminated leishmaniasis (DL), and post‐kala‐azar dermal leishmaniasis (PKDL) forms [1]. Parasite persistence following apparent clinical resolution of the initial lesion is often associated with lesion recurrence, systemic dissemination, and progression to more severe disease phenotypes. These outcomes are influenced by both intrinsic characteristics of the parasite and the host’s immune response.

*Leishmania amazonensis* (*L. amazonensis*) is notably associated with CL, DCL, DL, and, in some instances, VL [2, 3]. A key mechanism facilitating the survival and dissemination of *L. amazonensis* amastigotes is the apoptotic mimicry, wherein viable parasites expose phosphatidylserine (PS) on their surface to mimic apoptotic cells. This strategy enables their recognition and uptake by host phagocytes via efferocytosis pathways [4, 5]. PS recognition by macrophages promotes amastigote internalization via macropinocytosis and suppresses nitric oxide production by induction of anti‐inflammatory cytokines such as interleukin‐10 (IL‐10) and transforming growth factor‐β (TGF‐β) [4]. Furthermore, PS signaling impairs dendritic cell activation, inhibiting the expression of MHC class II molecules, CD86, IL‐1β, and IL‐12, thereby reducing the cells’ ability to prime effective T‐cell responses [5].

In mouse models of intradermal infection, systemic treatment with anti‐PS antibodies reduces the parasite load and enhances antigen‐specific T‐cell activation [5]. In patients with DCL, PS exposure on the surface of *L. amazonensis* parasites correlates positively with disease duration and the number of disseminated lesions, indicating a link between PS‐mediated immune modulation and disease severity [5, 6]. Despite these findings, the role of PS in parasite dissemination and visceralization remains incompletely understood.

The intradermal ear infection model in mice is a well‐established in vivo system for studying *Leishmania* pathogenesis as it closely mimics natural infection via sandfly bite. In BALB/c mice, which are highly susceptible to the infection, parasites initially establish localized lesions and migrate to the draining lymph nodes by approximately 5 weeks postinfection (p.i.). However, systemic dissemination is limited, typically resulting in low parasite burdens in visceral organs [7]. Models capable of evaluating systemic dissemination in the context of CL‐causing species are scarce but could yield important insights into the immunopathogenesis of cutaneous and disseminated disease manifestations.

## 2. Material and Methods

### 2.1. Mice and Parasite

Female BALB/c mice aged 6–8 weeks were used in all experiments. Animals were housed at the animal facility of the Polo IMCT – Universidade Federal do Rio de Janeiro (UFRJ), Macaé, Brazil, under controlled environmental conditions (22–25°C), with free access to food and water (ad libitum). All experimental procedures were conducted in accordance with institutional ethical guidelines and were approved by the Ethics Committee on Animal Use of the UFRJ, Campus Macaé (Protocol Number MAC047). *Leishmania amazonensis* parasites (strain MPRO/BR/72/M1845) were used throughout this study.

### 2.2. Amastigote Isolation and Purification

Amastigotes were obtained from the infected footpads of female BALB/c mice according to the protocol described by Wanderley (2019). Briefly, infected footpad tissues were aseptically excised and mechanically homogenized using a glass Potter‐type tissue homogenizer in cold DMEM. The homogenate was centrifuged at 50 × *g* for 10 min at 4°C to remove tissue debris. The resulting supernatant was carefully collected and centrifuged at 1750 × *g* for 17 min at 4°C to pellet the amastigotes. The pellet was resuspended and washed twice in DMEM. The parasite suspension was then incubated for 2 h at 34°C with gentle agitation (50 rpm) in DMEM supplemented with 4% fetal bovine serum (FBS) to promote recovery and remove residual host cell debris. Subsequently, amastigotes were washed twice and incubated overnight at 34°C in DMEM containing 4% FBS. After incubation, parasites were centrifuged, washed three times in sterile DMEM, and maintained in DMEM supplemented with 4% FBS at 4°C until use. To confirm parasite purification and assess viability, amastigotes were stained with Annexin V and propidium iodide (PI) to evaluate PS exposure and membrane integrity according to the manufacturer’s instructions.

### 2.3. Intravenous Infection of Mice With Amastigotes and Anti‐PS Antibody Treatment

BALB/c mice were intravenously inoculated via the tail vein with 1 × 10^6^ *Leishmania amazonensis* amastigotes suspended in sterile PBS. The parasite burden in target organs was assessed weekly using a limiting dilution assay. For PS blockade experiments, purified amastigotes were incubated in vitro with 5 µg/mL of anti‐PS monoclonal antibody (clone PGN635, Creative Biolabs, #MOB‐066LC) for 30 min at 4°C. Two antibody formats were used for amastigote coating, according to the experimental group: the full‐length antibody (11:31) and fragment antigen‐binding (Fab). Following antibody incubation, parasites were intravenously inoculated into mice, as described above. 4 weeks p.i., animals received intraperitoneal administration of PBS or the same anti‐PS antibody format used for amastigote coating. Mice treatment consisted of intraperitoneal injections of 100 µg of antibody administered twice weekly for three consecutive weeks according to the experimental group defined at the time of parasite inoculation. At 12 weeks p.i., animals were euthanized, and tissues were collected for subsequent analyses of parasite burden and dissemination. The data represent two independent experiments. Ten individual animals were included per experimental group, resulting in a total of 60 mice across both experiments.

### 2.4. Evaluation of Intracellular Cytokine Expression in Splenocytes

Cytokine production was evaluated by intracellular flow cytometry using splenocytes obtained from each individual mouse in a total of 10 mice per experimental group. Cells were stimulated with 50 µg/mL of soluble leishmanial antigen (SLA) for 72 h. During the final 4 h of incubation, a leukocyte activation cocktail containing BD GolgiPlug (BD Biosciences) was added to the culture medium to allow intracellular accumulation of cytokines. After incubation, the cells were centrifuged at 700 × *g* for 5 min, and the supernatant was discarded. The cell pellet was resuspended in PBS containing 2% paraformaldehyde for 15 min. Cells were then centrifuged and resuspended in PBS containing 0.1% saponin and 2% normal mouse serum for an additional 15 min to reduce nonspecific binding. Subsequently, cells were incubated with fluorochrome‐conjugated monoclonal antibodies for 30 min and diluted in PBS containing 0.1% saponin and 2% normal mice serum. The antibody panel included CD3‐FITC, CD11b‐PE, IL‐4‐PE‐Cy7, IL‐10‐PE, TGF‐β‐PerCP‐Cy5.5, and TNF‐α‐APC. After staining, cells were washed with perm‐wash buffer, resuspended in PBS, and analyzed by flow cytometry. The experimental procedure was adapted from Bottrel et al. (2001). Data acquisition and analysis were performed using FlowJo CE software (Cytek).

### 2.5. Analysis of Lesion Distribution and Size

The lesion distribution and size were assessed macroscopically. Distribution was categorized based on lesion location using the following scoring criteria: 0: no apparent ulcer; 1: one ulcer on the tail; 2: one ulcer on the paw; and 3: ulcers on both paws or on the tail and one paw. The lesion size was scored as follows: 0: no visible ulcer; 1: small ulcer (0–5 mm); 2: medium‐sized ulcer (6–10 mm); and 3: large ulcer (>10 mm).

### 2.6. Statistical Analysis

Results were expressed as the mean ± standard deviation (SD). Data normality was assessed using the Shapiro–Wilk test. For normally distributed data, two‐way analysis of variance (ANOVA) was performed, followed by Tukey’s test for multiple comparisons. For nonnormally distributed data, the Kruskal–Wallis test was used. Differences were considered statistically significant at a *p* ≤ 0.05. All analyses were conducted using GraphPad Prism 9 software (GraphPad Software, USA).

## 3. Results and Discussion

### 3.1. Establishment of a Systemic Model for *L. amazonensis* Dissemination

To investigate the role of PS in *L. amazonensis* dissemination, we established a systemic infection model using intravenous inoculation of amastigotes and evaluated parasite visceralization, immune responses, and cutaneous dissemination in mice treated with anti‐PS antibodies. First, BALB/c mice were injected intravenously with 1 × 10^6^ amastigotes, and the parasite burden was monitored weekly in multiple organs. Parasites were first detected in the bone marrow during the first week p.i. and persisted throughout the 12‐week observation period. Dissemination to the spleen occurred by week 7 p.i., followed by involvement of inguinal and mesenteric lymph nodes at week 8 and the liver at week 9. All organs maintained detectable parasite loads until week 12 p.i. when the experiment concluded (Figure 1A).

**Figure 1:**
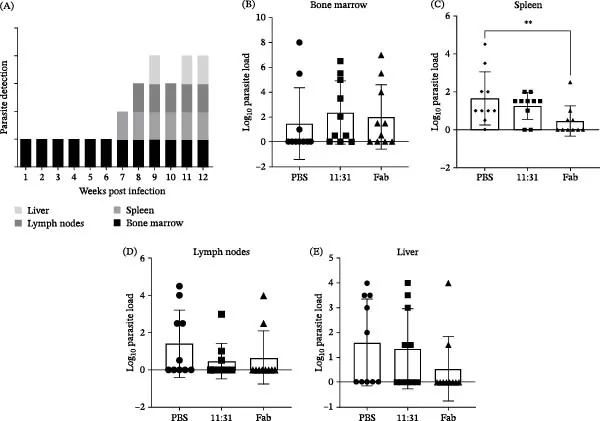
Antiphosphatidylserine (anti‐PS) antibodies reduce visceral parasitism in a systemic *L. amazonensis* infection model. BALB/c mice were intravenously infected with 1 × 10^6^ *L. amazonensis* amastigotes in 100 μL of PBS. (A) Parasite burden in the spleen, liver, bone marrow, mesenteric, and inguinal lymph nodes derived from four mice per week was monitored over a 12‐week period using a limiting dilution assay following 15 days of axenic culture. (B–E) Mice were treated with 100 μg of anti‐PS full antibody (clone 11:31) or their Fab fragments intraperitoneally, three times per week from week 4 to week 7 postinfection. Parasite loads were assessed at 12 weeks postinfection in the (B) bone marrow, (C) spleen, (D) lymph nodes, and (E) liver. Data are presented as mean ± SD from groups of 10 mice. Statistical significance was determined using two‐way ANOVA with Kruskal–Wallis post‐test.  ^∗∗^ *p*  < 0.01.

These results confirm that intravenous inoculation of viable amastigotes provides a reproducible model for systemic infection, reproducing the main features of visceral dissemination and persistence. Early colonization of the bone marrow agrees with previous observations that dermotropic *Leishmania* species persist in the marrow and spleen during chronic infection [8]. In *L. braziliensis*, parasites are detected in the spleen, bone marrow, and lymph nodes within 6 weeks p.i., whereas viscerotropic species such as *L. donovani* infect the liver first, followed by the spleen and bone marrow [9]. The bone marrow’s sinusoidal vasculature, rich in migrating leukocytes, may facilitate parasite transport by infected host cells [8]. The delayed lymph node parasitism observed here suggests that hematogenous spread precedes lymphatic dissemination.

### 3.2. Anti‐PS Treatment Reduces Visceral Parasite Load

To determine the contribution of PS to parasite dissemination, we employed the full‐length anti‐PS monoclonal antibody 11:31 and its Fab fragments, which have previously been shown to have therapeutic potential in cancer, viral, and parasitic diseases [5, 10]. Mice were infected with amastigotes coated with either 11:31 or Fab antibodies and received intraperitoneal antibody injections three times per week for 3 weeks, beginning 4 weeks p.i. At 12 weeks p.i., the splenic parasite burden was significantly reduced in Fab‐treated animals compared with PBS controls (Figure 1C). The liver and lymph node parasite load also declined in both anti‐PS‐treated groups, although these differences did not reach statistical significance (Figure 1D, E). No significant difference in the parasite burden was observed in the bone marrow between control and treated mice (Figure 1B).

Parasites of the genus *Leishmania* expose PS on their surface, a mechanism associated with immune evasion known as apoptotic mimicry. By exposing PS, parasites mimic apoptotic cells and exploit host phagocytic pathways, facilitating their internalization by phagocytic cells and promoting an anti‐inflammatory response that favors parasite survival. Blocking PS has previously been associated with reduced parasite burden both in vitro [11] and in dermal lesions of C57BL/6 mice [5]. Our results reinforce the idea that PS neutralization can interfere with the progression of *Leishmania* infection, including in a systemic context.

In this study, we evaluated two antibody formats: the full‐length antibody and the Fab fragment. The full‐length antibody is composed of both Fab and Fc regions. While the Fab region is responsible for antigen recognition, the Fc region can interact with Fcγ receptors (FcγR) expressed on immune cells, triggering several effector mechanisms such as phagocytosis, antibody‐dependent cell‐mediated cytotoxicity (ADCC), production of reactive oxygen species, lysosomal enzyme release, and immune complex formation. Therefore, although the full antibody may block PS recognition, the interaction between the Fc portion and FcγR may potentially promote the internalization of opsonized parasites by phagocytic cells [12] or modulation of cytokine production by macrophages [13], which could explain the lack of a significant reduction in parasite burden in this group.

The Fab fragment proved more effective than the intact antibody, likely due to its improved tissue penetration and absence of Fc‐mediated effects [14]. In contrast, the intact antibody retains an Fc region that can act as an opsonin, enhancing parasite uptake through FcγR‐bearing macrophages and potentially promoting infection [13]. The superior efficacy of the Fab fragment, therefore, supports a mechanism based primarily on neutralization of PS exposure rather than immune complex formation.

### 3.3. PS Blockade Modulates Immunoregulatory Cytokine Responses

Because *L. amazonensis* infection induces a mixed Th1/Th2 immune profile that permits chronicity; we next assessed whether PS blockade modulated systemic cytokine production. Splenocytes were collected at week 12 p.i. Cells were stimulated with SLAs, and intracellular cytokine expression was analyzed by flow cytometry after GolgiPlug. A schematic overview of the flow cytometry gating strategies is provided in Figure S1 (Figure S1). Fab‐treated mice displayed significant reductions in IL‐10 and TGF‐β production by CD3^+^ T cells (Figure 2A,), suggesting attenuation of regulatory cytokine dominance. No significant changes were observed in IL‐4 expression by CD3^+^ T cells (Figure 2C) or TNF‐α expression by CD11b^+^ myeloid cells (Figure 2D). IL‐10, TGF‐β, and nitric oxide production by myeloid cells were not significantly altered (data not shown).

**Figure 2:**
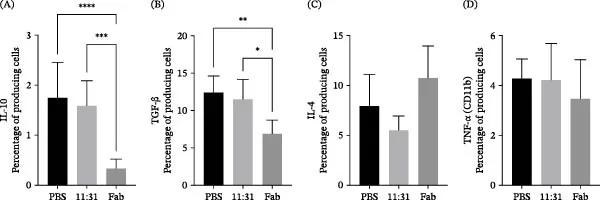
Anti‐PS antibody treatment reduces IL‐10 and TGF‐β production by CD4^+^ T cells during systemic *L. amazonensis* infection. BALB/c mice were intravenously infected with 1 × 10^6^ *L. amazonensis* amastigotes coated with anti‐PS full antibody (clone 11:31) or Fab fragments. Mice received 100 μg of the respective antibody intraperitoneally, three times per week from week 4 to week 7 postinfection. At 12 weeks postinfection, splenocytes were harvested and cultured with soluble leishmanial antigens for 72 h. During the final 6 h, PMA, ionomycin, and monensin were added to assess intracellular cytokine production by flow cytometry. Cytokine expression was evaluated in (A) IL‐10 and (B) TGF‐β in CD3^+^ T cells, (C) IL‐4 in CD3^+^ T cells, and (D) TNF‐α in CD11b^+^ myeloid cells. Statistical analysis was performed using two‐way ANOVA with Kruskal–Wallis post‐test.  ^∗^ *p*  < 0.05;  ^∗∗^ *p*  < 0.01;  ^∗∗∗^ *p*  < 0.001;  ^∗∗∗∗^ *p*  < 0.0001.

PS exposure on apoptotic cells naturally triggers anti‐inflammatory signaling, inhibiting macrophage activation and promoting the release of IL‐10 and TGF‐β by these cells. *L. amazonensis* amastigotes exploit this apoptotic mimicry, displaying PS to suppress host immunity and favor chronic infection [15]. The observed reduction in IL‐10 and TGF‐β upon PS blockade demonstrates the reversal of this immunomodulatory advantage. In *L. donovani* infection, IL‐10 promotes susceptibility and splenic persistence [16], while IL‐10/TGF‐β‐producing Tregs contribute to chronicity in *L. infantum* [17]. Interestingly, IL‐10 produced by regulatory CD8^+^ T cells inhibits nitric oxide production during *L. guyanensis* cutaneous‐localized infection [18]. Conversely, these cytokines can limit tissue injury in cutaneous diseases [19]. Since we evaluated cytokine production by CD3^+^ T cells, both CD4^+^ and CD8^+^ populations could contribute to the regulatory phenotype observed in infected hosts. The differences observed in cytokine production between antibody formats reinforce that the immunological changes detected are primarily associated with PS blockade by the Fab fragment rather than immune complex formation. It is also important to note that the absence of significant changes in the groups treated with the full‐length antibody may be related to the recognition of immune complexes formed with opsonized amastigotes. In *Leishmania mexicana*, a dermotropic species closely related to *L. amazonensis*, IgG bound to the parasite surface can be recognized by FcγR, inducing IL‐10 production [20]. This mechanism may explain the lack of reduction in IL‐10 production in the group treated with the full antibody.

Therefore, PS blockade may differentially affect visceral and dermal manifestations by shifting the balance away from regulatory suppression toward protective responses.

### 3.4. PS Blockade Limits Cutaneous Dissemination

To evaluate whether systemic PS blockade altered disease progression, macroscopic skin lesions were assessed at week 12 p.i. as indicators of parasite dissemination. Lesions appeared on the tails and paws of infected mice, excluding the injection site. Treatment with Fab anti‐PS antibodies significantly reduced both lesion size and frequency, whereas full‐length 11:31 antibodies limited lesion dissemination but did not significantly affect lesion size (Figure 3A–C). Cutaneous lesions distant from the inoculation site represent dissemination driven by hematogenous spread and local inflammatory reactivation [21–24]. The marked reduction in lesion size and distribution following Fab treatment likely reflects both decreased visceral parasite load and reduced macrophage infection in distal sites. The differences observed between the two antibody formats further support the conclusion that the effects described here are primarily attributable to PS recognition blockade. The findings obtained with the full‐length antibody also reinforce previous studies showing that opsonization represents one of the strategies used by *Leishmania* amastigotes to infect host cells [12].

**Figure 3:**
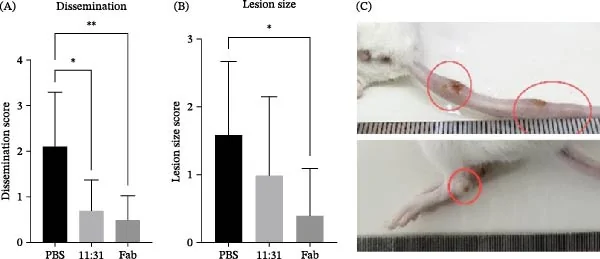
Anti‐PS antibody treatment limits the development of disseminated skin lesions in systemically infected mice. BALB/c mice were intravenously infected with 1 × 10^6^ *L. amazonensis* amastigotes and treated with 100 μg of anti‐PS full antibody (clone 11:31) or Fab fragments intraperitoneally three times per week from week 4 to week 7 postinfection. At 12 weeks postinfection, the presence of disseminated skin lesions was assessed macroscopically. (A) Lesion distribution and (B) lesion size were scored based on a standardized scoring system. (C) Representative images of lesion severity. Data are expressed as mean ± SD. Statistical significance was determined by two‐way ANOVA with Kruskal–Wallis post‐test.  ^∗^ *p*  < 0.05.

These data indicate that PS blockade not only restricts parasite persistence in visceral organs but also mitigates peripheral reactivation, leading to decreased pathology.

## 4. Concluding Remarks

Altogether, these results demonstrate that the 11:31 anti‐PS antibody—particularly its Fab fragment—effectively restricts systemic *L. amazonensis* infection. The lower efficacy of the full antibody likely results from Fc‐mediated uptake, a known entry route for amastigotes [20]. The observed decline in IL‐10 and TGF‐β production correlates with reduced parasitism in visceral organs and decreased dissemination to distal cutaneous sites. By interfering with PS‐dependent immunoregulatory pathways, Fab anti‐PS treatment restores host immune competence, countering a major evasion mechanism exploited by *L. amazonensis*. Given the extensive PS exposure on the surface of amastigotes, this molecule likely constitutes a key virulence factor underlying the broad clinical spectrum and persistence of disease caused by this species. Future work integrating histopathological and immunophenotypic analyses of disseminated lesions in this model will clarify how PS orchestrates macrophage signaling, T‐cell regulation, and tissue tropism in progressive leishmaniasis.

## Funding

This work was supported by the CAPES/Brazil (Grant 88882.449607/2019‐01) and the Fundação de Amparo à Pesquisa do Rio de Janeiro (Grants E‐26/210.118/2022 and E‐26/204.136/2024).

## Conflicts of Interest

The authors declare no conflicts of interest.

## Supporting Information

Additional supporting information can be found online in the Supporting Information section.

## Supporting information


**Supporting Information** Figure S1: Representative flow cytometric analysis of cytokine intracellular staining. BALB/c mice were intravenously infected with 1 × 10⁶ L. amazonensis amastigotes coated with anti‐PS full antibody (clone 11:31) or Fab fragments. Mice received 100 μg of the respective antibody intraperitoneally, three times per week from week 4 to week 7 postinfection. At 12 weeks postinfection, splenocytes were harvested and cultured with soluble leishmanial antigens for 72 h. During the final 6 h, PMA, ionomycin, and monensin were added to assess intracellular cytokine production by flow cytometry. Suspended and adherent cells were harvested and evaluated. (A) Dot plot analysis of forward and side scatter and gating strategy. (B) CD3‐FITC staining and gating strategy to select T lymphocytes. (C) Dot plot analysis of CD3^+^ cells intracellularly stained for IL‐10 PE. (D) Dot plot analysis of CD3^+^ cells intracellularly stained for IL‐4 PE‐Cy7. (E) Dot plot analysis of CD3^+^ cells intracellularly stained for TGF PerCP‐Cy5.5. (F) Dot plot analysis of CD3‐CD11b+ cells intracellularly stained for TNFα APC.

## Data Availability

Data are available for sharing.
